# Association of pre-pregnancy low-carbohydrate diet with maternal oral glucose tolerance test levels in gestational diabetes

**DOI:** 10.1186/s12884-022-05059-2

**Published:** 2022-09-26

**Authors:** Yanhui Hao, Lei Qu, Yuna Guo, Liying Ma, Muhe Guo, Yiqing Zhu, Yan Jin, Qin Gu, Yue Zhang, Wenguang Sun

**Affiliations:** 1 International Peace Maternity and Child Health Hospital, Shanghai Jiao Tong University, 910 Hengshan Road, 200030 Shanghai, China; 2 Shanghai Key Laboratory of Embryo Original Diseases, Shanghai, 200030 China; 3 Department of Nutrition, School of Medicine, Shanghai Jiao Tong University, Shanghai, 200092 China

**Keywords:** Gestational diabetes mellitus, Dietary pattern, Pregnancy, Oral glucose tolerance test, Low-carbohydrate diet

## Abstract

**Background:**

Limited evidence exists on the correlation between the pre-pregnancy low-carbohydrate (LC) diet and maternal oral glucose tolerance test (OGTT) levels during pregnancy. Our aim was to compare the differences in maternal OGTT levels among women who had been diagnosed with gestational diabetes mellitus (GDM) during pregnancy and adopted different dietary patterns in the pre-pregnancy period.

**Methods:**

A case–control study was conducted in 20 women with GDM who adhering to an LC diet (carbohydrate intake < 130 g/d) during pre-conception (LC/GDM,cases). Control subjects, who were matched in a 4:1 ratio, were 80 women with GDM and conventional diet (Con/GDM,control), and 80 women with conventional diet but without GDM (Con/Healthy,control). Women diagnosed with GDM using 75-g OGTT between 24 and 28 weeks of gestation. We used unadjusted raw data to compare the dietary composition data and biomarkers of the three study groups.

**Results:**

The average pre-conception BMI in each group suggested a similar body size from the three study groups(19.12 ± 2.00 LC/GDM, 19.65 ± 2.32 Con/GDM, 19.53 ± 2.30 Con/Healthy; *P* = 0.647). Compared with the Con/GDM group, the OGTT-1 h and OGTT-2 h values in LC/GDM group were significantly higher (10.36 ± 1.28 mmol/L vs. 9.75 ± 0.98 mmol/L; 9.12 ± 0.98 mmol/L vs. 8.29 ± 1.06 mmol/L). Furthermore, the percentage of women who had more than one abnormal OGTT value (OGTT-1 h and OGTT-2 h) was 40% in the LC/GDM group, which was significantly higher than in the Con/GDM group (16.3%).

**Conclusions:**

We observed a relationship between the pre-pregnancy LC diet and more detrimental OGTT values in patients with GDM. This finding warrants further studies to understand the effect of pre-pregnancy LC diet practice on maternal glucose tolerance.

**Supplementary Information:**

The online version contains supplementary material available at 10.1186/s12884-022-05059-2.

## Introduction

Gestational diabetes mellitus (GDM), characterized by glucose intolerance first diagnosed during pregnancy, is one of the most common maternal complications [1]. In China, a recent systematic review and meta-analysis including 79,064 participants showed a total incidence of 14.8%, while the Shanghai Birth Cohort found 14.2% (585 women) with GDM [2, 3]. Epidemiologic data have shown that GDM is associated with both short- and long-term adverse health consequences for the mother and the offspring [4]. Risk factors of developing GDM include those with advanced maternal age, pre-pregnancy overweight/obesity, family history of diabetes, and excessive weight gain during pregnancy [5]. Poor dietary behaviors before and during pregnancy, such as excessive consumption of sugary drinks and higher intake of animal fat and cholesterol, were also associated with an increased risk of GDM [6].

Regarding the optimum diet for a gestational diabetic woman, Jovanovic-Peterson et al. suggested that the primary treatment strategy for pregnancies complicated by diabetes should be one which does not precipitate ketones of starvation, but restricts carbohydrates sufficiently to prevent postprandial hyperglycemia in 1990 [7, 8]. In 2014, Bao et al. found that, compared with the highest pre-pregnancy carbohydrate intake group, the lowest pre-pregnancy carbohydrate intake group had a 27% higher risk of GDM (relative risk [RR] = 1.27, 95% CI: 1.06, 1.51) [9]. The Australian Longitudinal Study on Women’s Health’s results also suggested that relatively low-carbohydrate (LC) diet and high fat intakes may increase the risk of GDM [10]. However, the above studies compared the differences between the lowest (lowest quintile of carbohydrate score) and highest (highest quintile of carbohydrate score) carbohydrate intake groups based on the LC diet scores rather than by absolute intakes of carbohydrate, fat, and protein; the lowest carbohydrate intakes were 178 g/d and 162 g/d, respectively; Low-carbohydrate diet, a dietary pattern with carbohydrate restriction, is a popular approach in weight loss and glucose control [11]. The dietary guidelines for Chinese residents (2022) recommends at least 130 g/d of carbohydrate intake during early pregnancy, while the Institute of Medicine (IOM) recommends at least 175 g/d of carbohydrate intake during pregnancy [12]. For the purposes of our study, we refer to diets with less than 130 g/day as a low-carbohydrate (LC) diet, consistant with the definition in recent reviews on safety of low carbohydrate diets [13, 14]. Despite the increasing popularity of strictly LC diets for weight loss in women of childbearing age, but it remains unknown whether the LC-diet is safe for maternal metabolic needs over longer period of time.

The purpose of this case–control study was to evaluate the differences in OGTT levels between women with GDM adhering to a LC diet (LC/GDM) and a conventional diet (Con/GDM) during pre-conception, and healthy pregnant women adhering to a conventional diet (Con/Healthy) during pre-conception for healthy control.

## Methods

### Study design and participants

This case–control study was conducted at Shanghai International Peace Maternity and Child Health Hospital (IPMCH) between January 2019 and January 2020. According to the routine medical procedures for pregnant women in Shanghai, a 75-g oral glucose tolerance test (OGTT) will be arranged between 24 and 28 weeks’ gestation to detect GDM. Women with a diagnosis of GDM will be referred to nutrition clinic for nutritional consultation and treatment. All participants were recruited after OGTT and dietary assessment between 24 and 28 weeks’ gestation.

**Fig. 1:**
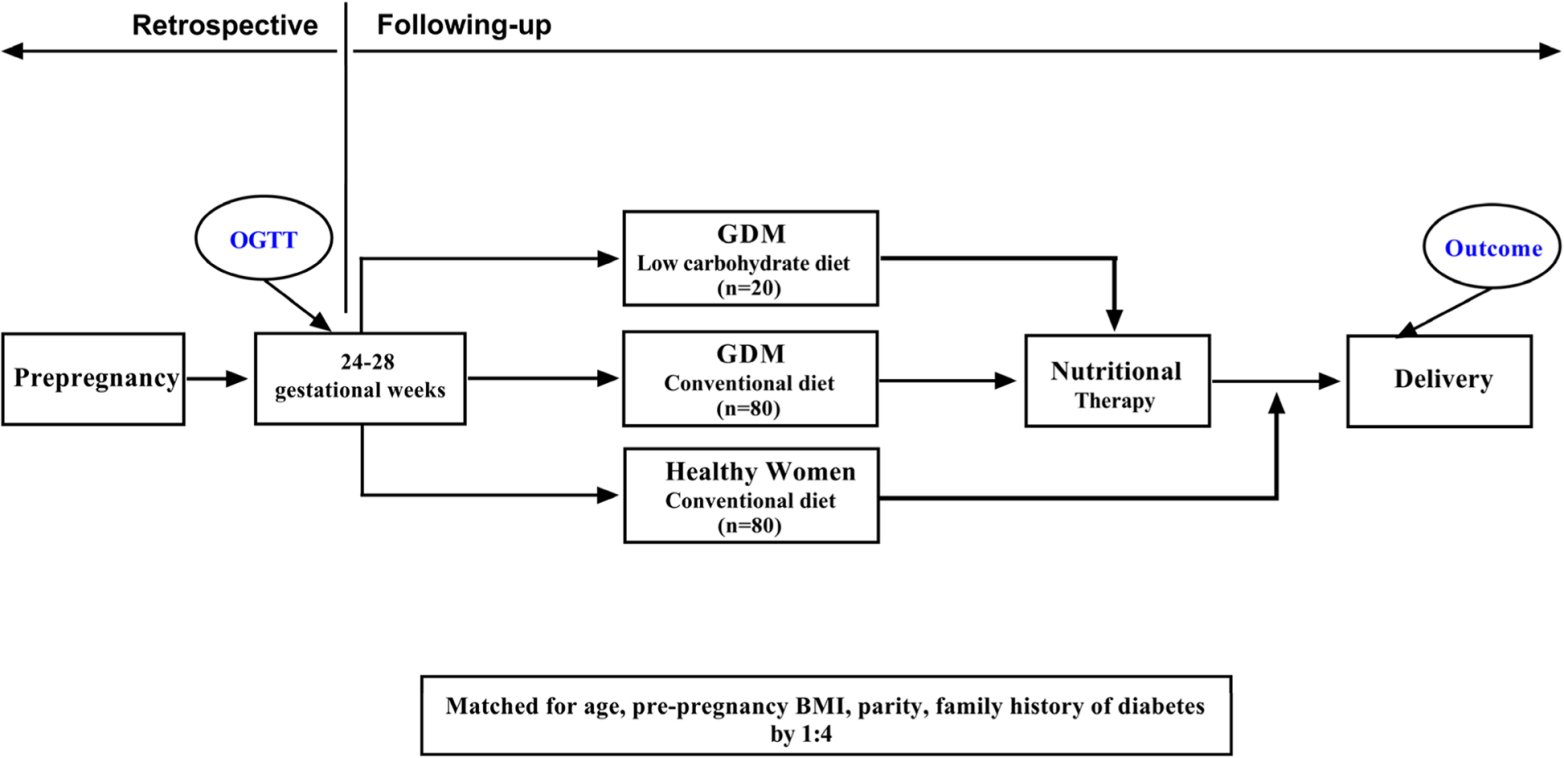
Patient workflow

- The participants recruited in the “case” group (LC/GDM) were women with a diagnosis of GDM, and the women with GDM were further interviewed with trained dietitians to identify if she had adhering to an initiative limit-carbohydrate diet (carbohydrate intake < 130 g/d) for weight management for at least 12 months before pregnancy.
- The participants recruited in the first “control” group (Con/GDM) were women with a diagnosis of GDM, and the women with GDM were further interview with trained dietitians to identify if she had adhering to an conventional diet (carbohydrate intake ≥ 130 g/d) before pregnancy.
- The participants recruited in the second “control” group (Con/Healthy) were healthy women on a conventional diet (carbohydrate intake > 130 g/d) before pregnancy.
- The two control groups (Con/GDM and Con/Healthy) were matched to the LC/GDM group by 1:4 for age, pre-pregnancy BMI (underweight, normal, overweight, obese), parity and family history of diabetes. A total of 180 pregnant women in 24-28 gestational weeks were included in this study (Fig. 1).

Subjects with the following criteria were excluded: 1) those with religious dietary restrictions; 2) those diagnosed with type 2 diabetes mellitus (T2DM) before pregnancy;3) those with history of polycystic ovary syndrome, unexplained recurrent spontaneous abortion, and other digestive tract disease, liver disease, and kidney disease. Cut-off points of BMI were adopted based on the Chinese population standards [15].

Written informed consent was obtained from all participants. This study was conducted according to the guidelines laid down in the Declaration of Helsinki and all procedures involing human subjects/patients were approved by the Institutional Review Board of the IPMCH affiliated with the School of Medicine, Shanghai Jiao Tong University (GKLW2018-35).

### Dietary assessments

The women in the “case” group (LC/GDM) were healthy women before pregnancy, who followed a long-term (≧12 months) LC diet for weight management. During the nutritional interview, participants’ habitual dietary intake before pregnancy was varified through a 24-h dietary recall about the average intake of major foods or food groups by trained dietitians. The low-carbohydrate (LC) diet was defined as diets with less than 130 g/day, and a conventional diet (Con diet) was defined as diets with carbohydrate intake ≥ 130 g/d, and without special food restriction.

Participants were also invited to complete a 24-h dietary recalls on their current dietary intake (between 24 and 28 weeks of gestation). If a participant reported during a 24-h recall that she did not eat or drink normally the day before (e.g., because of fasting, illness, or other reasons), dietary data from that 24-h recall will be performed at another visit.

Trained dietitians administered the interview, using food models to access the food intake. The interviews were conducted between 24 and 28 weeks of gestation. The intakes of energy and macronutrients were calculated by nutrient calculation software ZHEN DIN *2.0*.

### OGTT value and diagnosis of GDM

Each OGTT value (fasting, 1 h, and 2 h) were compared among three groups. The type and number of abnormal OGTT values were also compared, such as the numbers (preportions) of women with an abnormal fasting value, abnormal 1-h value, or with an abnormal 2-h value. The “Higher-1 h-2 h” refer to women who had more than one abnormal OGTT value (OGTT-1 h and OGTT-2 h).

GDM was diagnosed by the International Association of the Diabetes and Pregnancy Study Groups (IADPSG) criteria between 24 and 28 weeks of gestation. A one-step approach using 75-g OGTT was performed after at least an 8-h fast (cutoff values: fasting ≥ 5.1 mmol/L; 1 h ≥ 10.0 mmol; 2 h ≥ 8.5 mmol/L). GDM was defined as one or more abnormal OGTT values [16].

Area under the curve (AUC) of OGTT were calculated according to the approximate trapezoidal area formula, AUC = (fasting blood glucose/2 + postprandial 1 h + postprandial 2 h/2) × 1 h[mmol/(L˙h)].

### Clinical examinations

All participants underwent standard clinical care which included biomedical blood tests. Fasting blood glucose, serum glycated haemoglobin (HbA1c) and lipid profiles [total cholesterol (TC), triacylglycerol (TG), high density lipoprotein cholesterol (HDLC), low density lipoprotein cholesterol (LDLC)]were measured in the first trimester of pregnancy (10–12 weeks of gestation). Serum HbA1c, glycated albumin (GA) and levels of OGTT test were performed between 24–28 gestational weeks. All the blood samples were detected with Cobas 8000 modular analyzer series. The detection of ketones in the urine was performed through Sysmex UC-3500 urine chemistry analyzer.

### Anthropometric measurements

The participants were weighed barefoot using a standardized digital height and weight scale calibrated to 0.1 kg (SECA-285). After resting for 5 min, an automated system (OMRON-HEM-1020) was used to measure the blood pressure once. Weighing and blood pressure measurements were performed during every routine check-up.

### Statistical analysis

Given the exploratory nature of this study (the sample size of women with a strict LC diet was limited), we did not conduct a priori sample size estimation. We only used unadjusted data to compare the dietary composition data and biomarkers of the three study groups because we have matched the potential confounders in different groups. One-way analysis of variance was used to analyze difference in continuous variables, and pairwise comparisons between the three groups were performed using Tukey’s studentized range test. Continuous variables were showed as means and SD. Chi-square test was used to analyze difference in categorical variables, and pairwise comparisons were performed using Bonferroni test. Statistical analysis was performed using SPSS statistical software, version 23. *P* values < 0.05 was considered statistically significant.

## Results

### Characteristics of the participants

The main characteristics of the study groups participants were presented in Table 1. As expected, all study participants were comparable in terms of age, parity, educational level, pre-pregnancy BMI, and family history of diabetes. There was no significant difference between the weight gains during pregnancy in the three groups in the first trimester. In the second trimester, the LC/GDM group had the lowest weight gain (5.72 kg) followed by the Con/GDM group (7.01 kg); the Con/Healthy group gained the most weight (8.33 kg). There was no difference in weight gain among women in the LC/GDM and Con/GDM groups in the third trimester, while the gestational weight gain in GDM groups were significantly lower than that in the Con/Healthy group (*P* < 0.001). No other significant differences were observed, including infant birth weight and macrosomia rate.

**Table 1:** Characteristics of the study population

	**LC/GDM (*N* = 20)**	**Con/GDM (*N* = 80)**	**Con/Healthy (*N* = 80)**	***p*-value**
**Maternal characteristics**				
**Maternal age(years)**				
Mean (SD)	31.4 ± 3.98	30.1 ± 3.38	31.1 ± 3.59	0.147
N (%)				1.000
≤ 35	19 (95)	76 (95)	76 (95)	
> 35	1 (5)	4(5)	4 (5)	
**Parity, n (%)**				1.000
Nulliparous	18 (90)	72 (90)	72 (90)	
Multiparous	2 (10)	8 (10)	8 (10)	
**Education, n (%)**				0.595
High school and lower	3 (15)	15 (18.75)	19 (23.75)	
College and above	17 (85)	65 (81.25)	61 (76.25)	
**Pre-pregnancy BMI (kg/m**^**2**^**)**				
Mean (SD)	19.1 ± 2.00	19.7 ± 2.32	19.5 ± 2.30	0.647
n (%)				1.000
< 18.5	10 (50)	40 (50)	40 (50)	
18.5–23.9	9 (45)	36 (45)	36 (45)	
≥ 24	1 (5)	4 (5)	4 (5)	
**Race, n (%)**				1.000
Han	20 (100)	78 (97.5)	77 (96.25)	
Non-Han	0 (0)	2 (2.5)	3 (3.75)	
**Family history of diabetes, n (%)**				1.000
NO	18 (90)	72 (90)	72 (90)	
Yes	2 (10)	8 (10)	8 (10)	
**Blood pressure**				
Systolic (mmHg)	107.8 ± 16.7	112.7 ± 11.1	109.2 ± 11.5	0.102
Diastolic (mmHg)	69.6 ± 12.9	70.6 ± 11.1	70.2 ± 10.0	0.923
**Lipid profile**				
TG (mmol/L)	1.28 ± 0.79	1.32 ± 0.50	1.21 ± 0.44	0.345
TC (mmol/L)	4.31 ± 0.94	4.57 ± 0.71^b^	4.19 ± 0.82	< 0.05
LDLC (mmol/L)	2.45 ± 0.81	2.46 ± 0.65	2.23 ± 0.75	0.104
HDLC (mmol/L)	2.19 ± 0.49	2.08 ± 0.49	1.99 ± 0.44	0.182
**Gestational weight gain**				
Gestational weeks (wk)	13.2 ± 3.0	12.5 ± 1.2	12.5 ± 0.9	0.125
1^st^ trimester	1.95 ± 2.72	2.18 ± 2.60	1.92 ± 2.32	0.798
Gestational week interval (wk)	16.4 ± 2.6	16.3 ± 1.5	16.3 ± 1.2	0.945
2^nd^ trimester	5.72 ± 2.08^a^	7.01 ± 2.41^b^	8.33 ± 2.56^c^	< 0.001
Gestational week interval (wk)	9.5 ± 1.5^a^	10.8 ± 1.2	10.8 ± 1.6^c^	< 0.05
3^rd^ trimester	3.11 ± 1.75	3.31 ± 2.30^b^	5.26 ± 2.54^c^	< 0.001
Gestational weeks (wk)	39.1 ± 0.7	39.2 ± 1.6	39.7 ± 1.6	0.072
Total weight gain	10.8 ± 3.93	12.5 ± 4.42^b^	15.5 ± 4.45^c^	< 0.001
**Preeclampsia, n (%)**				0.792
No	19 (95)	78 (97.5)	78 (97.5)	
Yes	1 (5)	2 (2.5)	2 (2.5)	
**Thyroid dysfunction**				0.904
**No**	19 (95)	75 (93.75)	74 (92.5)	
**Yes**	1 (5)	5 (6.25)	6 (7.5)	
**Mode of delivery**				0.102
Vaginal birth	6 (30)	47 (58.75)	42 (52.5)	
Cesarean section	14 (70)	33 (41.25)	38 (47.5)	
**Gestational age (weeks)**	39.1 ± 0.7	39.2 ± 1.6	39.7 ± 1.6	0.072
**Child characteristics**				
**Birth weight (g)**	3113.4 ± 289.5	3193.7 ± 493.4	3267.9 ± 471.3	0.354
**LGA, n (%)**	0 (0)	4 (5)	5 (6.25)	0.534
**SGA, n (%)**	0 (0)	2 (2.5)	1 (1.25)	0.690

*LC* Low carbohydrate diet, *GDM* Gestational diabetes mellitus, *Con* Conventional diet, *SD* Standard deviation, *TG* Triglyceride, *TC* Total cholesterol, *LDLC* Low-density lipoprotein-cholesterol, *HDLC* High-density lipoprotein-cholesterol, *LGA* Large-for gestational age, *SGA* Small-for gestational age

^a^The value for the LC/GDM group is significantly different from the value for the Con/GDM group (*P* < 0.05)

^b^The value for the Con/GDM group is significantly different from the value for the Con/Healthy group (*P* < 0.05)

^c^The value for the LC/GDM group is significantly different from the value for the Con/Healthy group (*P* < 0.05)

### Ditary intake

**Fig. 2:**
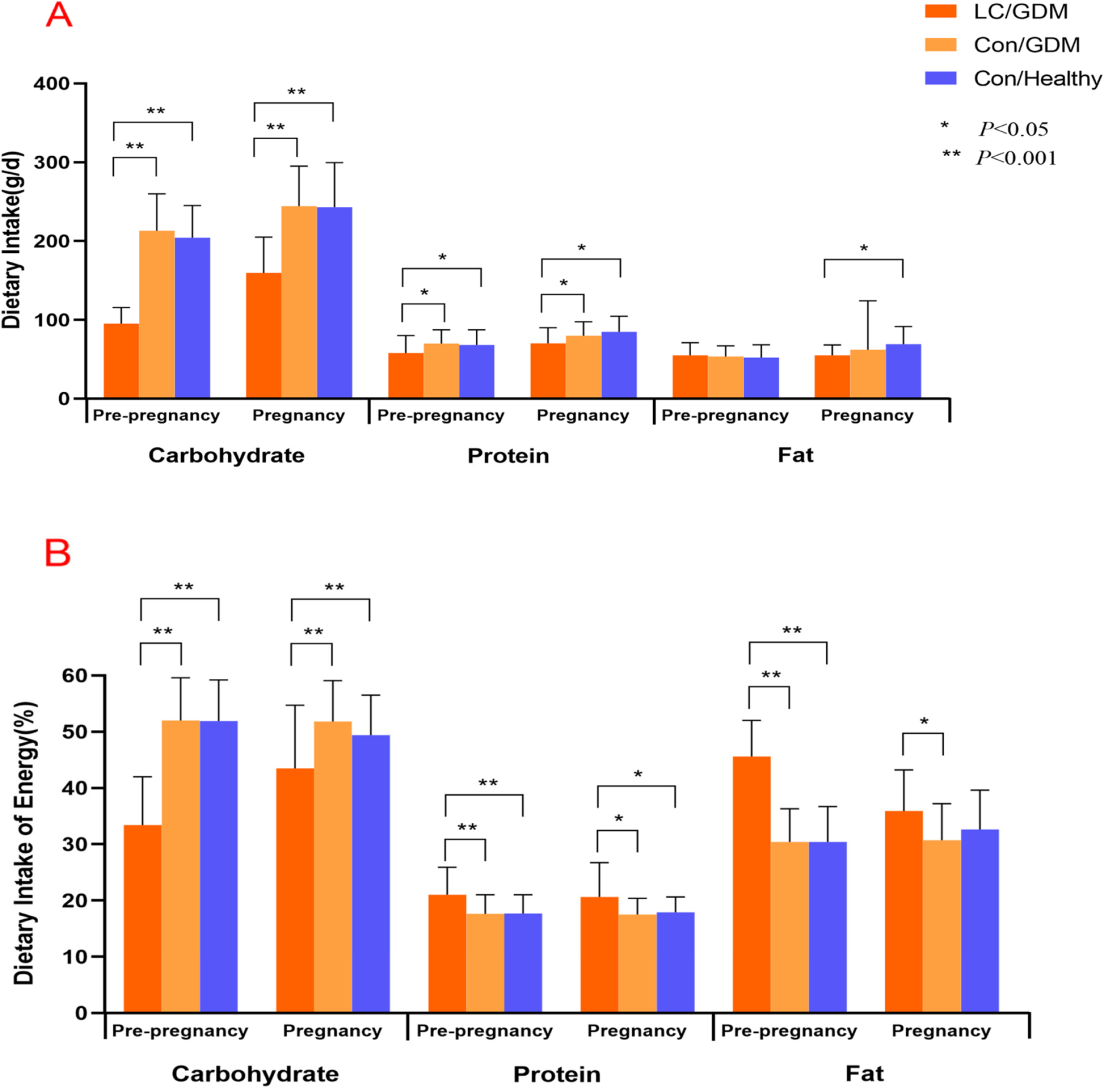
Changes in dietary intake pre-pregnancy and during the second trimester of pregnancy

Figure 2 shows the dietary intake data of the study groups before pregnancy and at the second trimester of pregnancy. The total energy intake before pregnancy in the LC/GDM group was 1078.5 kcal/d, which was significantly lower than the 1585.5 kcal/d in the Con/GDM group (*P* < 0.001) and 1532.5 kcal/d (*P* < 0.001) in the Con/Healthy group. During the second trimester, the energy intakes were increased in all three groups, however, women in the LC/GDM group still consumed much less calories(1375.6 ± 192.9 kcal) compared to the Con/GDM (1826.9 ± 268.3 kcal) and Con/Healthy (1899.2 ± 364.0) groups (*P* < 0.001).

The LC/GDM group had the lowest intake of carbohydrates (95.3 g/d; energy supply ratio, 33.4%) compared to the other two groups before pregnancy, with minimum and maximum intakes of 52.8 g/d and 126.6 g/d, respectively, which met the LC intake standard of < 130 g/d. All study groups showed increased carbohydrate intake during the second trimester. The intake in the LC/GDM and Con/GDM groups increased by an average of 64.4 g and 31.3 g, respectively. Although the LC/GDM group had the highest increase in carbohydrate intake, that in the second trimester was still lower than that in the other two groups (*P* < 0.001), and six (30%) participants in the LC/GDM group still followed an LC diet in the second trimester.

In the pre-pregnancy period, the protein intake level (58.1 g/d) of the LC/GDM group was lower than that of the Con/GDM group (69.9 g/d). The protein energy supply ratio of the LC/GDM group was 21%, which was higher than the 17.6% of the Con/GDM group in the pre-pregnancy period. There was no difference in the total fat intake among the three groups during the pre-pregnancy period (*P* = 0.729), but the fat energy supply ratio of the LC/GDM group was significantly higher than that of the Con/GDM and Con/Healthy groups (*P* < 0.001).

The dietary patterns of the Con/GDM and Con/Healthy groups were similar before and during pregnancy in terms of the energy and macronutrient intakes and ratio, which were consistent with the recommended range of the Chinese food pagoda [17]. Compared with the Con/GDM and Con/Healthy groups, the LC/GDM group followed a typical LC diet before pregnancy.

### Fasting plasma glucose and OGTT

Compared with women in the Con/GDM group, those in the LC/GDM group had a lower HbA1c level in the first trimester (5.14% vs. 5.32%, *P* < 0.05). Compared with the first trimester, the fasting blood glucose level of the three groups in the second trimester showed a downward trend (Table 2).

**Table 2:** Changes in biomarkers according to the diet group, with or without gestational diabetes mellitus

	**LC/GDM (*N* = 20)**	**Con/GDM (*N* = 80)**	**Con/Healthy (*N* = 80)**	***p*-value**
**Variable**				
**1**^**st**^** trimester of pregnancy**				
Gestational weeks(wk)	11.98 ± 2.16	11.93 ± 1.96	12.41 ± 1.53	0.222
FBG (mmol/L)	4.41 ± 0.43	4.50 ± 0.35	4.57 ± 0.33	0.165
HbA1c (%) (mmol/mol)	5.14 ± 0.46 (33) ^a^	5.32 ± 0.27 (35) ^b^	5.19 ± 0.32 (33)	< 0.05
**2nd trimester of pregnancy**				
Gestational weeks(wk)	25.51 ± 1.11	25.28 ± 1.20	25.20 ± 1.00	0.539
OGTT				
Fasting (mmol/L)	4.15 ± 0.45	4.19 ± 0.48	4.11 ± 0.33	0.411
1 h (mmol/L)	10.4 ± 1.28^a^	9.75 ± 0.98^b^	7.15 ± 1.16^c^	< 0.001
2 h (mmol/L)	9.12 ± 0.98^a^	8.29 ± 1.06^b^	6.19 ± 1.01^c^	< 0.001
Abnormal values, type				
Fasting (≥ 5.1 mmol/L [92 mg/dL]) (n, %)	1 (5)	5 (6.25)	Reference	0.064
1 h (≥ 10.0 mmol/L [180 mg/dL) (n, %)	14 (70)	43 (53.75) ^b^	Reference	< 0.001
2 h (≥ 8.5 mmol/L [153 mg/dL) (n, %)	14 (70)	49 (61.25) ^b^	Reference	< 0.001
Abnormal values, n (%)				
0	0(0)	0(0)	80(100)	< 0.001
≧1(GDM)	20(100)^a^	80(100) ^b^	0(0)	< 0.001
1	11(55)^a^	64(80) ^b^	0(0)^c^	< 0.001
2	9(45)^a^	15(18.75) ^b^	0(0)^c^	< 0.001
3	0(0)	1(1.25)	0(0)	0.556
Higher-1 h-2 h (n, %)	8 (40)^a^	13 (16.25) ^b^	0 (0)^c^	< 0.001
AUC [mmol/(L˙h)]	17.0 ± 1.13^a^	16.0 ± 0.92^b^	12.6 ± 1.85^c^	< 0.001
HbA1c (%) (mmol/mol)	4.90 ± 0.30 (30)	5.05 ± 0.35 (31) ^b^	4.92 ± 0.26 (30)	< 0.05
GA (%)	12.31 ± 1.23^a^	13.8 ± 1.59	13.49 ± 1.34^c^	< 0.001
Insulin treatment, n (%)				< 0.001
Yes	2 (10) ^a^	0 (0)	0 (0) ^c^	< 0.001
**Detectable urinary ketone bodies** (n, % of participants)				
1^st^ trimester of pregnancy	2 (10)	2 (2.5)	5 (6.25)	0.306
2^nd^ trimester of pregnancy	5 (25) ^a^	1 (1.25)	1 (1.25)^c^	< 0.001
3^rd^ trimester of pregnancy	1 (5)	1 (1.25)	6 (7.5)	0.158

*LC* Low carbohydrate diet, *GDM* Gestational diabetes, *Con* Conventional diet, *FBG* Fasting blood glucose, *HbA1c* Glycated haemoglobin, *OGTT* Oral glucose tolerance test, *AUC* Area under the blood glucose curve, *GA* Glycated albumin

^a^The value for the LC/GDM group is significantly different from the value for the Con/GDM group (*P* < 0.05)

^b^The value for the Con/GDM group is significantly different from the value for the Con/Healthy group (*P* < 0.05)

^c^The value for the LC/GDM group is significantly different from the value for the Con/Healthy group (*P* < 0.05)

**Fig. 3:**
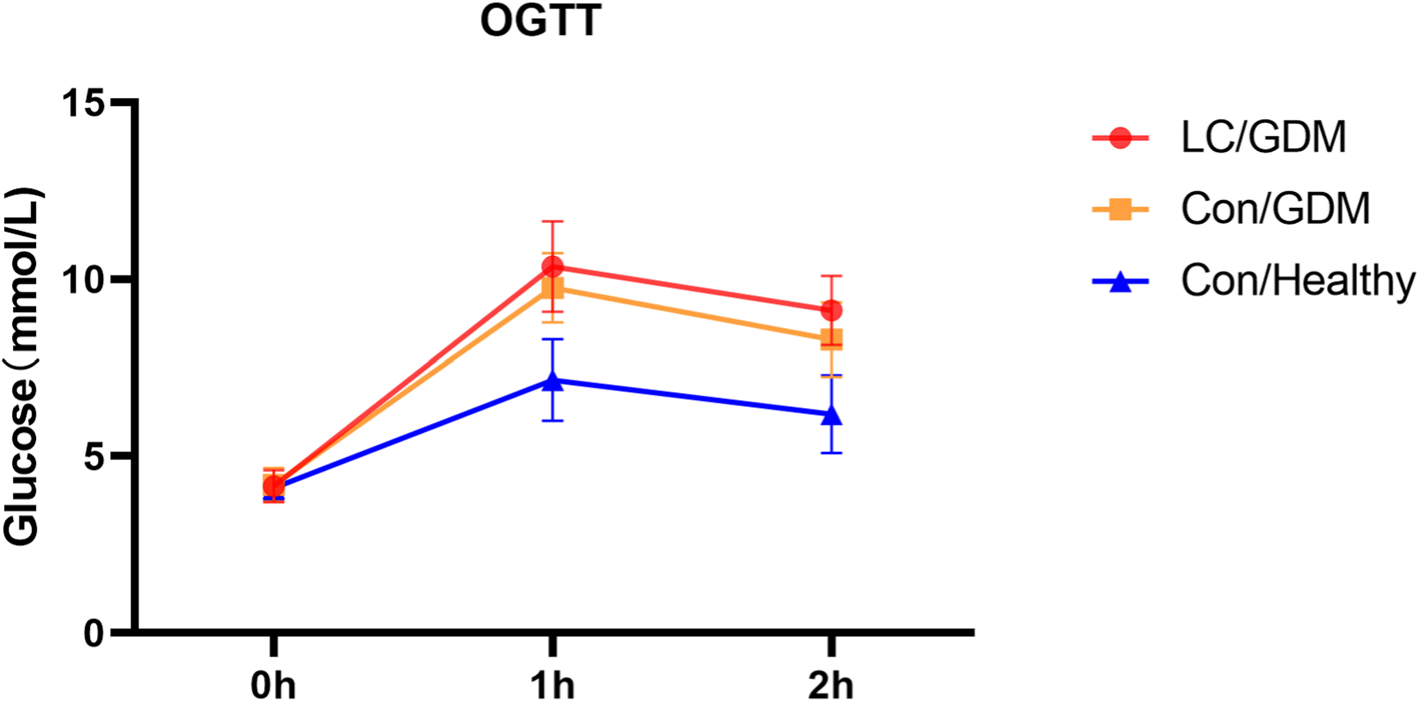
The blood glucose levels of the two groups of participants diagnosed with GDM and the healthy controls at the three time points of the OGTT. LC, low carbohydrate diet; GDM, gestational diabetes; OGTT, oral glucose tolerance test. LC/GDM, patients with GDM on an low carbohydrate diet; Con/GDM, patients with GDM on a conventional diet; Con/Healthy, healthy women on a conventional diet

For comparing the blood glucose levels among the three groups at the three OGTT timepoints (OGTT-0 h, OGTT-1 h, and OGTT-2 h), the OGTT-0 h blood glucose levels in the LC/GDM and Con/GDM groups were not significantly different. However, those at OGTT-1 h and OGTT-2 h in the LC/GDM group were significantly higher than those in the Con/GDM group. The area under the blood glucose curve was also significantly higher than that of the Con/GDM group (*P* < 0.001) (Fig. 3). In addition, compared with the Con/GDM group, the ratio of abnormal values in both OGTT-1 h and OGTT-2 h in the LC/GDM group was significantly higher (LC/GDM: 40.0%; Con/Healthy: 16.3%). In the first and second trimesters, the HbA1c levels in the LC/GDM and Con/GDM groups were the lowest and highest, respectively, among the three groups. There was no significant difference in HbA1c levels between the LC/GDM and Con/GDM groups in the second trimester. However, in the second trimester, glycated albumin (GA) of the LC/GDM group was significantly lower than that of the Con/GDM group (*P* < 0.001).

There was no significant difference in the rate of positive urine ketone bodies among the three groups in the first trimester. In the second trimester, the rate in the LC/GDM greatly increased, which was significantly higher than that in the Con/GDM and Con/Healthy groups (*P* < 0.001). In the third trimester, the positive rate of positive urine ketone bodies in LC/GDM group decreased to 5%.

## Discussion

We found that compared with women with GDM who had a conventional diet before pregnancy, those who adhered to a long time LC diet before pregnancy had not only higher values in OGTT-1 h and OGTT-2 h but also higher rate of abnormal values both in OGTT-2 h and OGTT-2 h. This study is the first to explore the relationship between a strict LC diet before pregnancy and glucose tolerance during pregnancy. First, the daily carbohydrate intake of the LC/GDM group were less than 130 g, which belong to strict LC diet [13, 18]. Second, different from studies showing that LC diets were mostly for medical treatment, such as weight loss in obese people and therapy for patients with type 2 diabetes [19, 20], participants in this study were healthy women before pregnancy, who followed a long-term (≧12 months) LC diet to control body mass. To our best knowledge, both Bao’s study in 2014 and Looman’s study in 2018 had suggested that pre-pregnancy LC diet may increase the risk of GDM. The lowest quintile of carbohydrate intakes in these studies were 178 g/d and 162 g/d, respectively. Although the intake of 162 g of carbohydrate/day were taking in less than the recommended 175 g/d, it was higher than the carbohydrate intakes in the LC group in our study [9, 10]. 

Studies had suggested that the higher the OGTT glucose levels were, the more severe the imparied glucose tolerance [21]. While the GDM patients with a LC diet (LC/GDM) showed a more severe impaired glucose tolerance than the GDM with a conventional diet (Con/GDM), our study also indicated that long-term LC diet before pregnancy may be associated with impaired glucose tolerance during pregnancy. The possible explanation was that long-term LC diets make the body mobilize fat for energy, and lipolysis produces more free fatty acids (FFAs) which may lead to insulin resistance (or aggravates pregnant insulin resistance). Although effects of LC diet on maternal FFAs remain unclear [22], Hales’s study on healthy men showed a significant increase in OGTT glucose values and serum free fatty acids (FFAs) after 5 days of LC diet [23]. Hernandez’s study in 2014 show the concentration of FFAs in the LC diet group (carbohydrates 40%, fat 45%, protein 15%) was significantly higher than that of the high-carbohydrate diet group (carbohydrates 60%, fat 25%, protein 15%) [24]. Existing mechanistic studies also confirmed that the increase in serum FFAs can cause lipotoxicity through endoplasmic reticulum stress [25], reactive oxygen species [26], apoptosis, and inflammatory response [27], which leads to insulin resistance [28, 29]. Another possible explanation was degenerated function of pancreatic islet because of long-term low carbohydrate load in the LC group. Our study showed that HbA1c levels in the LC/GDM group were 5.14% (33 mmol/mol) and 4.90% (30 mmol/mol) in the first and the second trimesters, respectively, which were the lowest among the three groups. The HbA1c levels suggested that the participants in the LC/GDM group might had a long-term low carbohydrate load. Animal studies reported in 2014 showed that a reduction in β-cell mass was observed in mice on long-term LC diet (22 weeks) [30]. Our ongoing animal experiments also found that an LC diet for 4 weeks led to a decrease in the number of pancreatic islet β-cells in mice (to be reported separately).

To support the growth of the fetus in the second trimester, the intakes of total energy and carbohydrate in the LC/GDM group increased compared with those before pregnancy, but there were still six participants whose carbohydrate intakes less than 130 g/d. When carbohydrate intake is low, the body consumes its own adipose tissue as energy, which may produce ketone bodies.

Carbohydrate restriction may also promote maternal ketonemia promoting oxidation of FFA to betahydroxybutyrate and other ketones [22]. This could be the reason that the rate of positive urine ketone bodies in the LC/GDM group was 25% in the second trimester, which was significantly higher than that in the other two groups. According to the current GDM diagnosis and treatment guidelines in IPMCH [31], women dignosed with GDM were given medical nutritional care, the participants in the LC/GDM were advised to increase their carbohydrate intake appropriately, we observed the rate of positive urine ketone bodies was decreased to 5% in the third trimester.

Notably, the Hyperglycemia and Adverse Pregnancy Outcome Follow-up Study (HAPO FUS) found the higher OGTT levels were associated with adverse outcome, such as macrosomia, cesarean delivery, neonatal hypoglycemia [32]. Furthermore, Hiersch et al. reported the more abnormal OGTT points during pregnancy, the higher the risk of type 2 diabetes after delivery [33]. Nishikawa et al. also found that the higher the level of OGTT-1 h, the higher the risk of impaired glucose tolerance after delivery [34]. Therefore, the abnormal glucose metabolism during pregnancy found in the LC/GDM population is particularly worthy of attention, and it provides a warning about the future risk of type 2 diabetes in the population with an LC diet. These findings highlight the urgent need for large-population, long time follow-up cohort studies on the long-term health effects of LC diets.

This study was designed to explore the relationship between a strict LC diet before pregnancy and glucose tolerance during pregnancy. Moreover, all the study participants were included during the similar time period, the food profiles were comparable among them. Different groups were matched by age, pre-pregnancy BMI, parity, conception, family history of diabetes and fasting blood glucose in first trimester of pregnancy to minimize the influence of confounding factors. However, there are several limitations to this study. First, the study sample size of the LC diet group was relatively small; however, given that the participants were a specific group of pregnant women, and based on the strict LC diet inclusion criteria, these inevitably limited the number of eligible women. Therefore, to fully compare the groups, we included a GDM conventional diet control group (Con/GDM group) and a healthy control group (Con/Healthy group) with good comparability. Second, assessment of diets is one of the most challenging behavioral assessments. Self-reporting and 24-h recall were combined to review the dietary patterns of the study participants before pregnancy. However, there may be recall bias.

## Conclusion

In this study, we observed higher values in OGTT-1 h and OGTT-2 h and higher rate of abnormal values both in OGTT-2 h and OGTT-2 h in the LC/GDM group, compared with the Con/GDM group. The data suggested a relationship between the pre-pregnancy low-carbohydrate diet and imparied glucose tolerance during pregnancy. This finding suggests that it is questionable whether women of childbearing age should adopt a LC diet to control weight. It also warrants further studies to understand the effect of pre-pregnancy low-carbohydrate diet behavior on imparied glucose tolerance and its underlying pathophysiology.

## Supplementary Information

**Additional file 1:** **STable 1.** Recommended range of macronutrient intakes from Chinese Dietary Reference Intakes. **STable 2.** Carbohydrate intake habit of LC/GDM group

## Data Availability

The datasets used and/or analysed during the current study are available from the corresponding author on reasonable request.

## Abbreviations

LC: Low-carbohydrate
OGTT: Oral glucose tolerance test
GDM: Gestational diabetes mellitus
LC/GDM: GDM women with the low-carbohydrate diet
Con/GDM: GDM women with the conventional diet
Con/Healthy: Women with conventional diet but without GDM
IPMCH: The International Peace Maternity and Child Health Hospital
IADPSG: The International Association of the Diabetes and Pregnancy Study Groups
HbA1c: Glycated haemoglobin
TC: Total cholesterol
TG: Triacylglycerol
HDLC: High density lipoprotein cholesterol
LDLC: Low density lipoprotein cholesterol
LGA: Large-for gestational age
SGA: Small-for gestational age
FBG: Fasting blood glucose
AUC: Area under the blood glucose curve
GA: Glycated albumin
SD: Standard deviation
FFAs: Free fatty acids
