# Supplementary material for: Association of pre-pregnancy low-carbohydrate diet with maternal oral glucose tolerance test levels in gestational diabetes
Source: BMC Pregnancy Childbirth. 2022 Sep 26;22:734. doi: 10.1186/s12884-022-05059-2 (PMC9511732; doi:10.1186/s12884-022-05059-2)
Supplement: Supplementary file 1 — Additional file 1: STable 1. Recommended range of macronutrient intakes from Chinese Dietary Reference Intakes. STable 2. Carbohydrate intake habit of LC/GDM group [file 12884_2022_5059_MOESM1_ESM.docx]

**STable 1. Recommended range of macronutrient intakes from Chinese Dietary Reference Intakes**

|  | **Pre-pregnancy** |  | **Pregnancy** | |
| --- | --- | --- | --- | --- |
|  |  |  | **Non-GDM** | **GDM** |
| ***Variable*** |  |  |  |  |
| Energy（kcal/d） | 2100~2300 |  | +200 | 1800~2200 |
| Carbohydrate (g/d) | - |  | >130 | >130 |
| Carbohydrate (% of energy) | 50 ~ 65 |  | 50 ~ 60 | 50~60 |
| Protein (g/d) | 65 |  | +0, +15, +30 | +0, +15, +30 |
| Protein (% of energy) | 10~15 |  | 15~20 | 15~20 |
| Fat (g/d) | - |  | - | - |
| Fat (% of energy) | 20 ~ 30 |  | 20 ~ 30 | 25~30 |
|  |  |  |  |  |

**STable 2. Carbohydrate intake habit of LC/GDM group**

|  | **Pre-pregnancy** | | |  | **Pregnancy** | | |
| --- | --- | --- | --- | --- | --- | --- | --- |
| ID | **Breakfirst** | **Lunch** | **Dinner** |  | **Breakfirst** | **Lunch** | **Dinner** |
| 1 |  √ | √ | × |  | √ | √ | × |
| 2 | Δ | Δ | Δ |  | √ | Δ | Δ |
| 3 | √ | √ | × |  | √ | √ | Δ |
| 4 | √ | √ | × |  | √ | √ | × |
| 5 | × | Δ | × |  | √ | √ | √ |
| 6 | √ | √ | × |  | √ | √ | Δ |
| 7 | Δ | Δ | Δ |  | √ | √ | Δ |
| 8 | √ | × | × |  | √ | × | × |
| 9 | × | Δ | Δ |  | × | Δ | Δ |
| 10 | √ | × | × |  | √ | × | × |
| 11 | √ | × | × |  | √ | × | Δ |
| 12 | × | × | × |  | × | Δ | × |
| 13 | √ | √ | × |  | √ | √ | × |
| 14 | × | Δ | × |  | × | Δ | Δ |
| 15 | Δ | Δ | × |  | √ | Δ | Δ |
| 16 | √ | Δ | Δ |  | √ | √ | Δ |
| 17 | × | √ | Δ |  | Δ | √ | Δ |
| 18 | Δ | Δ | × |  | √ | √ | Δ |
| 19 | Δ | Δ | × |  | √ | Δ | Δ |
| 20 | √ | Δ | Δ |  | √ | Δ | Δ |

√ Consume carbohydrates as needed.

× No carbohydrate intake initiatively .

Δ Control carbohydrate intake initiatively.

* The Con/GDM group and the Con/Healthy group consume carbohydrates as needed.
